# Loss of miR-204 expression is a key event in melanoma

**DOI:** 10.1186/s12943-018-0819-8

**Published:** 2018-03-09

**Authors:** Marco Galasso, Carl Morrison, Linda Minotti, Fabio Corrà, Carlotta Zerbinati, Chiara Agnoletto, Federica Baldassari, Matteo Fassan, Armando Bartolazzi, Andrea Vecchione, Gerard J. Nuovo, Gianpiero Di Leva, Stefania D’Atri, Ester Alvino, Maurizio Previati, Brian J. Nickoloff, Carlo M. Croce, Stefano Volinia

**Affiliations:** 1 LTTA, Department of Morphology, Surgery and Experimental Medicine, University of Ferrara, 44121 Ferrara, Italy; 2 Roswell Park Cancer Institute, Department of Pathology, Buffalo, NY 14263 USA; 3 Department of Medicine (DI-MED), Surgical Pathology Unit, University of Padua, 35121 Padua, Italy; 4 Pathology Research Laboratory, Sant’Andrea University Hospital, 00189 Rome, Italy; 5 University of Salford, Salford, M5 4WT UK; 6 Istituto Dermopatico dell’Immacolata, 00167 Rome, Italy; 7 Institute of Translational Pharmacology, National Council of Research, Rome, Italy; 8 Eli Lilly, Indianapolis, IN USA; 9 Department of Molecular Virology, Immunology and Medical Genetics and Comprehensive Cancer Center, Ohio State University, Columbus, OH 43210 USA

**Keywords:** Melanoma, Somatic alterations, microRNA, Non coding rna, Breslow, Melanocyte, Keratinocyte, BRAF, NRAS, CDKN2A

## Abstract

Cutaneous melanoma (CM) is a malignancy with increasing occurrence. Its microRNA repertoire has been defined in a number studies, leading to candidates for biological and clinical relevance: miR-200a/b/c, miR-203, miR-205, miR-204, miR-211, miR-23b and miR-26a/b. Our work was aimed to validate the role of these candidate miRNAs in melanoma, using additional patients cohorts and in vitro cultures. miR-26a, miR-204 and miR-211 were more expressed in normal melanocytes, while miR-23b, miR-200b/c, miR-203 and miR-205 in epidermis and keratinocytes. None of the keratinocyte-related miRNAs was associated with any known mutation or with clinical covariates in melanoma. On the other hand, the loss of miR-204 was enriched in melanomas with NRAS sole mutation (Fisher exact test, *P* = 0.001, Log Odds = 1.67), and less frequent than expected in those harbouring CDKN2A mutations (Fisher exact test, *P* = 0.001, Log Odds − 1.09). Additionally, miR-204 was associated with better prognosis in two independent melanoma cohorts and its exogenous expression led to growth impairment in melanoma cell lines. Thus, miR-204 represents a relevant mechanism in melanoma, with potential prognostic value and its loss seems to act in the CDKN2A pathway, in cooperation with NRAS.

**Electronic supplementary material:**

The online version of this article (10.1186/s12943-018-0819-8) contains supplementary material, which is available to authorized users.

## Results and discussion

### Key microRNAs in melanoma and normal epidermis

In previous studies some microRNAs have been shown to play a role in CM: miR-200a/b/c, miR-203 and miR-205 were negatively associated with tumor progression and have been proposed to slow down cell replication, migration and invasion in vitro*,* inhibiting anchorage-independent colony formation and epithelial-mesenchymal transition (EMT). [1, 2] Furthermore, miR-204, miR-205, miR-211, miR-23b and miR-26a/b were highly expressed in nevi [3] and miR-211 was proposed to enable a tumor suppressive function by itself or via its host gene, TRPM1 [1, 4–7]. On the opposite side, miR-211 is present in melanosomes produced by melanocytes, and it could increase melanoma invasiveness by the activation of MAPK in cancer-associated fibroblasts [8].

**Fig. 1:**
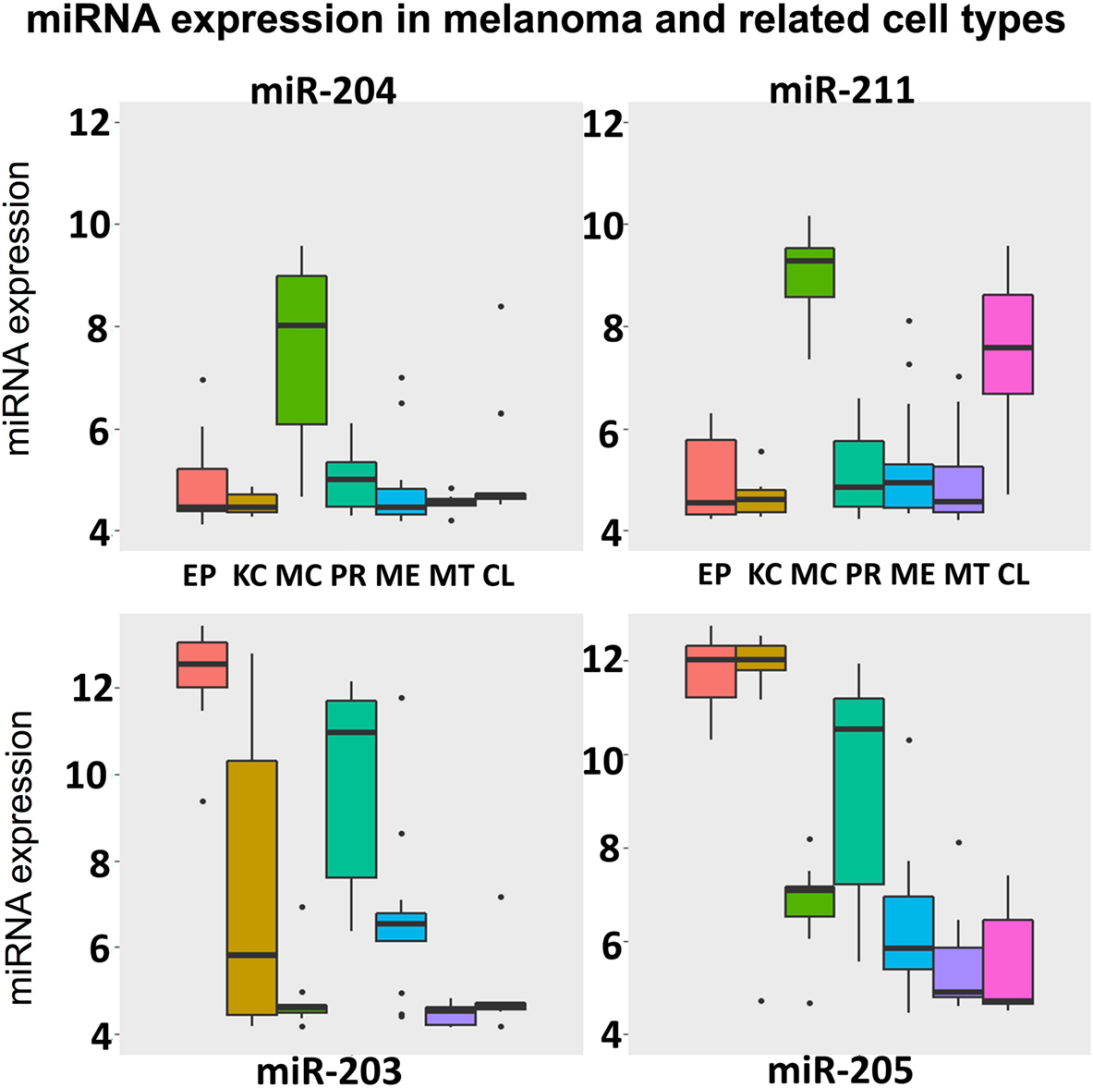
miRNA expression in melanoma and related cell types. Box-plots of miRNA expression are shown for candidate miRNAs (OSUMC microarrays). The y axis displays the quantiles normalized expression levels (log 2 scale); the different cell types are aligned on the x axis. The first three categories on the left are normal controls: epidermis (EP), cultured keratinocytes (KC) and cultured melanocytes (MC). The melanoma subgroups follow, in an order related to cancer progression: primary tumors (PR), melanoma metastasis (ME), low passage number (MT) and high passage number (CL) metastatic melanoma cell lines

Our work was aimed to re-evaluate and validate these candidate miRNAs, using additional patients cohorts and in vitro cultures. Eighty samples, including 15 pairs of matched primary/metastatic tumours, 12 normal skin biopsies, 11 cultured melanocytes, 10 cultured keratinocytes, and 17 melanoma cell lines were analyzed on miRNA microarrays, as previously reported microRNA OSU microarrays [9]. The table with quantile normalized miRNA expression (log2 RPM) is reported in Additional file 1. We identified 157 highly variable miRNAs (Additional file 2: Figure S1). Among the candidate miRNAs, miR-204 and miR-211 were significantly more expressed in cultured melanocytes than in keratinocytes and epidermis (Fig. 1 and Additional file 2: Figure S1), but much less in primary tumours and metastasis, in agreement with previous reports [1, 3, 5, 7]. On the opposite, miR-23b, miR200b/c, miR-203 and miR-205 [10] were significantly more expressed in epidermis and cultured keratinocytes than in cultured melanocytes, but also detected at low levels in primary melanoma samples. miR-26a had only 2-fold higher levels in melanocytes than in keratinocytes, and 2-fold lower in melanoma samples. In order to validate and extend our findings we needed and independent melanoma cohort, thus went on to further investigate all non-coding RNAs in the TCGA melanoma cohort (*n* = 452, Additional file 3: Table S1; http://firebrowse.org). The TCGA consortium described three mRNA-based subclasses in melanoma [11]: the so-called “immune”, “MITF-low” and “keratin”. Two major findings arose from working on the TCGA dataset. First, miR-200c, miR-203 and miR-205 were the three miRNAs having highest correlation with keratinocyte marker Keratin 5 (KRT5) mRNA (Pearson *r* = 0.68, *r* = 0.61 and *r* = 0.77, *P* < 1e-07) and miR-203/− 205 were sufficient to identify the samples belonging to the “keratin” subtype (Additional file 4: Figure S2); secondly, two markers used to identify melanoma cells, MITF and MLANA, showed no correlation with miR-200b/c, miR-203 and miR-205, while negatively associating with miR-204 (*r* = − 0.2 and *r* = − 0.21, both *P* < 0.001, respectively), and positively with miR-211 (*r* = 0.71 and *r* = 0.69, both *P* < 1e-07; Additional file 5: Table S2). To better comprehend these findings, we studied keratins in melanoma cell lines and patient-derived melanoma xenografts (PDXs) [12]: only few samples showed expression of KRT5, and at very low levels. Conversely, and as expected, MITF and MLANA were strongly expressed in all melanoma cell lines and PDXs (Additional file 6: Figure S3). Thus, miR-200c, miR-203 and miR-205 were likely due to keratinocytes present within melanoma biopsies, both in TCGA and in our human samples. Reassuringly, the remaining candidate miRNAs had low (miR-23a) or no correlation with KTR5 (miR-204, miR-211, miR-26b). Thus, MITF showed positive association with miR-211 but not with miR-204. This finding was in line with MITF transcriptional up-regulation of host gene TRPM1 that thus indirectly drives the expression of miR-211. Our results show that the detection of miR-200c, miR-203 and miR-205 in melanoma samples was likely due to presence of keratinocytes within the biopsies.

### The expression of candidate miRNAs and somatic melanoma mutations

**Fig. 2:**
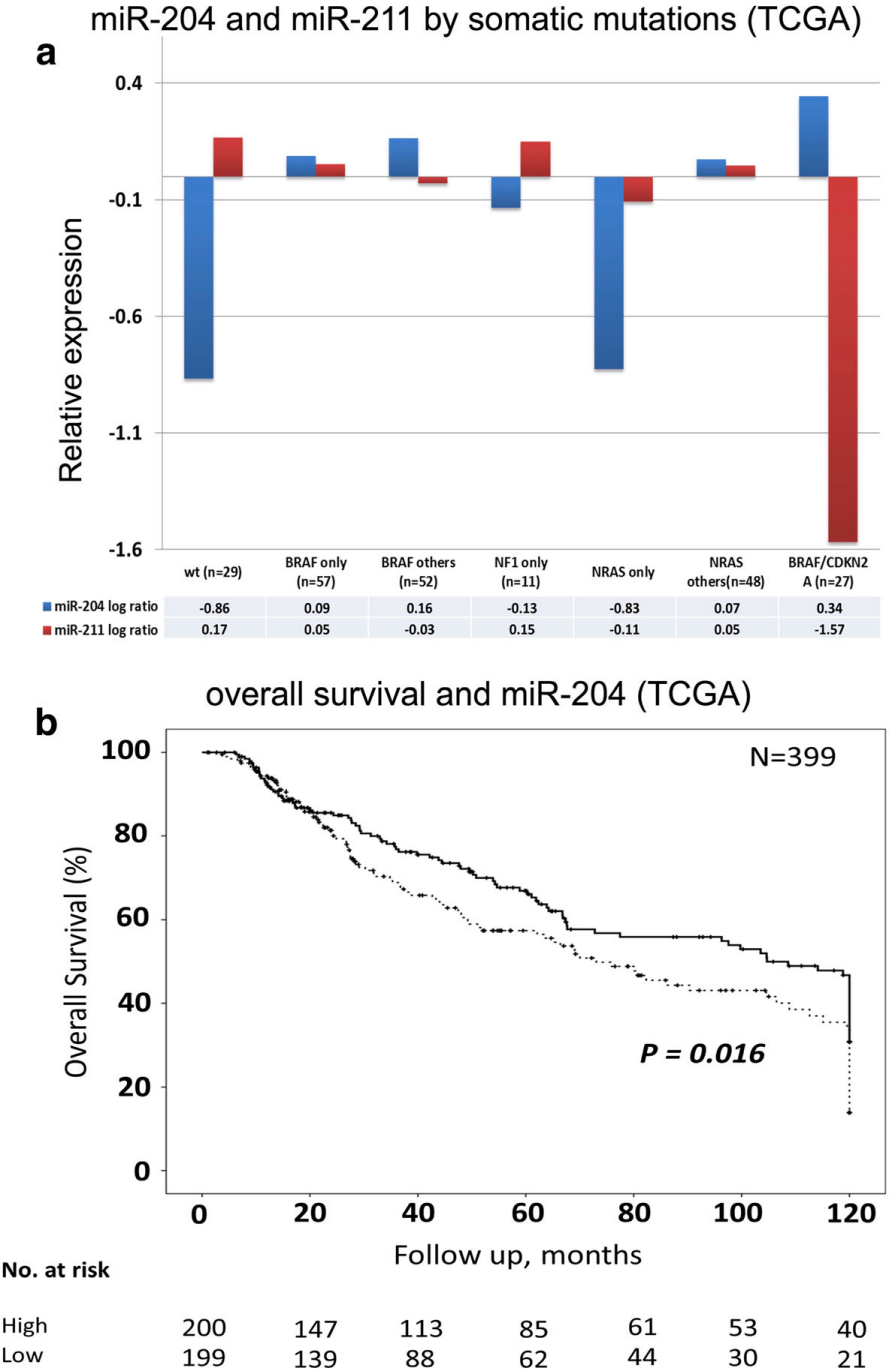
miR-204 expression in the melanoma mutation context and its association with overall survival. **a**) miR-204 and miR-211 log ratios plotted *(y-axes)* according to somatic mutation patterns. **b**) The Kaplan Meyer curve shows overall survival of melanoma patients divided according to miR-204 median level (continuous line: high miR-204; dashed line: low miR-204), in the TCGA cohort (*n* = 399, Log- Rank *P* < 0.01). The numbers of patients at risk are reported for each of the curves at the time range under the *x-axis* (expressed in months)

*BRAF* and *NRAS* are frequently somatically mutated in melanoma [11, 13, 14], along with NF1, CDKN2A (mutation and deletion), and CCND1 (amplification). Thus, we tested the miRNA expression in relation with these somatic alterations. Melanomas were split into 7 groups according to the most frequent mutation patterns: BRAF only (*n* = 57), sole NRAS (*n* = 37), NF1 only (*n* = 18), BRAF and CDKN2A (*n* = 59), non-sole NRAS (*n* = 42), remaining mutation patterns (*n* = 32), and no somatic events in any of the above-mentioned genes (*n* = 29) (Additional file 7: Table S3). Levels of miR-204 and miR-211 were strongly associated with somatic mutations, and with different trends (Additional file 8: Figure S4). The expression of miR-204 was lost in the sole NRAS mutated and in all wild-type tumours, while that of miR-211 was lower in the BRAF/CDKN2A double hits (Holm adjusted *P* < 0.05), as shown in Fig. 2A (see also Additional file 9: Figure S5). As controls miR-203, miR-205 and MITF RNA levels are invariant in the mutation subgroups (Additional file 10: Table S4). In particular, miR-204 loss was more frequent in sole NRAS than in non-sole NRAS (Fisher exact test, *P* = 0.001, Log Odds Ratio = 1.67). On the contrary, loss miR-204 expression was less frequent than expected in conjunction with homo-deleted or double compounded heterozygote CDKN2A when compared with wild type (Fisher exact test *P* = 0.001, Log Odds Ratio = − 1.09). The remaining miRNAs were not associated with any mutation group.

### The association of candidate miRNAs with clinical parameters in melanoma patients

We then investigated the prognostic potential of the candidate miRNAs. miR-204 was the only miRNA associated with prognosis in the TCGA cohort (*n* = 399), where its expression loss had a negative effect on survival (Additional file 7: Table S3). Figure 2B shows the Kaplan Meyer curve for miR-204 in overall survival (OS) for all patients (Cox regression odds ratio [OR], 0.71; 95% CI, 0.53–0.95 *P* = 0.02; Log Rank test on the median expression, *P* = 0.016). Furthermore, since miR-204 was down regulated in specific mutation subsets (Additional file 10: Table S4) we investigated its impact on survival in those contexts. The independent value of miR-204 was shown by a multivariate Cox regression, where the mutation subsets were confounding factors (Additional file 11). We validated the association of miR-204 with survival in an independent cohort of 32 melanoma samples (OSUMC), by using the TaqMan Array RT-PCR platform. The patients were divided in two groups by their median survival time (median OS = 7 years). The miR-204 expression was lower in samples with poor prognosis (*t-test*, *P* < 0.05), confirming the results of the TCGA cohort.

Finally, we studied the miRNAs in the Breslow’s stages, related with tumour invasion in the dermal layer. miR-204 and miR-211 expression correlated with Breslow’s thickness, with opposite, albeit not strong, trends: miR-211 was positively correlated (Spearman ρ = 0.2, *P* < 0.001, *n* = 309), while miR-204 had the negative correlation expected for its role on survival (ρ = − 0.13, *P* < 0.02, *n* = 309). Similar results were obtained using a trend-test rather than Spearman correlation (Additional Information). Among the miRNAs we evaluated in melanoma, only miR-204 and miR-211 were associated with important clinical parameters. Intriguing is the finding that miR-204 and miR-211 had an opposite relationship with Breslow’s stages. Our hypothesis is that melanoma cells expressing miR-204 and those expressing miR-211 belong to two subtypes with different invasive potential. This hypothesis is supported by the differences in mutation profiles we identified in the TCGA samples expressing the two miRNAs. Furthermore, a very recent work [15] shows that miR-204 is under the control of STAT3 and its expression is induced in amelanotic melanoma cells, where it can act as an effector of vemurafenib’s anti-motility activity. Conversely, miR-211 is induced in melanotic melanoma cells and serves as an effector of vemurafenib’s pro-pigmentation activity. It becomes thus more apparent a pro-invasiveness role for miR-211, as Dror et al. [8] described with melanosomal miR-211 directly targeting IGF2R and leading to MAPK signalling activation in cancer associated fibroblasts.

### miR-204 and miR-211 can inhibit growth in melanoma cell lines

**Fig. 3:**
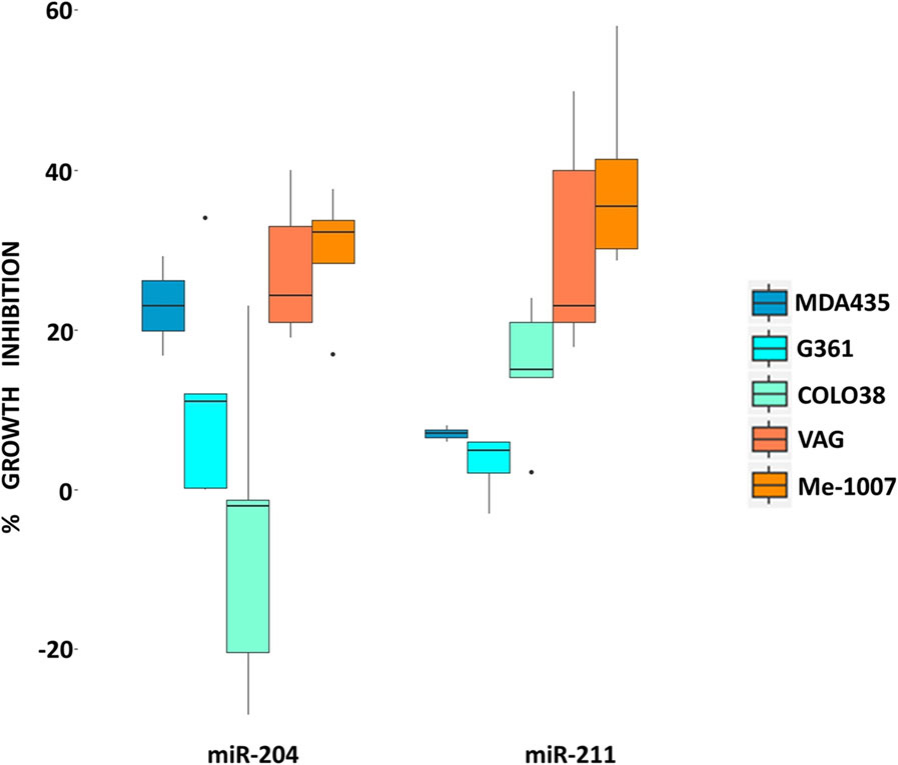
Exogenous miR-204 and miR-211 and cell growth in melanoma cell lines. The box-plots represent the relative cell growth inhibition upon transfection of miR-204 and miR-211 in five melanoma cell lines. MTS assays were baseline subtracted from control and normalized, as described in the supplementary methods (Additional file 15). The blue shaded cell lines were BRAF mutated and the orange were BRAF wild type. No cell line among those treated harbored an NRAS mutation

miR-204 and miR-211 share the same seed sequence, but their expression levels were inversely correlated between themselves (Pearson *r* = − 0.25, *P* < 1e-07) and with MITF and MLANA melanocytic markers. Furthermore miR-204 and miR-211 had opposite behaviours in somatic mutation groups, in Breslow’s depth and in prognosis. To study the miRNAs’ roles in vitro, we investigated their impact on five melanoma cell lines: MDA-MB-435, G361, VAG, Me-1007 and COLO38, differing for their BRAF and CDKN2A mutation patterns (Additional file 12: Table S7). miR-204 and miR-211 mimics were transiently transfected to assess their impact on cell viability (Fig. 3). VAG and Me-1007, harbouring no mutations in BRAF and CDKN2A, were sensitive to both miRNAs. On the contrary, doubly mutated G361 was insensitive to both miRNAs. Only miR-204 mimic inhibited MDA-MB-435, harbouring BRAF V600E and a CDKN2A splicing mutation, while only miR-211 inhibited Colo-38, a cell line mutated in BRAF but not in CDK2A. In summary, either miR-204 or miR-211 mimics inhibited cell growth in cell lines carrying wild type but not in those with a mutated BRAF (*t-test*, *P* < 0.01). In agreement with our findings, Diaz-Martinez and coworkers very recently showed that ectopic expression of both miRNAs was sufficient to augment in vivo tumor growth of A375 cells [16], that like G361 are BRAF/CDKN2A doubly mutated. These two related miRNAs share the same conserved seed, but their expression was inversely correlated when compared to melanocytic markers MITF and MLANA. miR-204 was significantly down-regulated in melanoma with wild type BRAF/NRAS or with sole NRAS mutation, as summarized in Additional file 13: Figure S6*.* In vitro, both miRNAs can inhibit growth of melanoma cell lines, and they seem to exert either equal (for example on BRAF/CDKN2A double wild type Me-1007 and VAG cell lines) or differential activity. It is possible that the common inhibition is exerted via the conserved seed, while the differential activity of the two miRNAs is due to the non-seed sequences. In fact, the miRanda database of predicted targets (http://microRNA.org at MSKCC), reports 3397 common predicted targets, 239 specific for miR-211 and 220 specific for miR-204 (Additional file 14: Table S8). On the other hand, when the two miRNAs are expressed in different mutation backgrounds, it is also possible that their effects, although mediated by the common seed, impact onto different targets. Of note, the loss of miR-204 expression was described in glioma, also thought to derive from a neural type precursor, where it led to an increase in cell migration and to a stem cell-like phenotype [17]. Furthermore, miR-204 was described as a key regulator of MMP9 and BCL2 in a xenograft model of melanoma [18].

## Additional files

Additional file 1: The table with quantile normalized miRNA expression (log2 RPM) of the eighty samples investigated. (XLSX 151 kb)

Additional file 2: **Figure S1.** Unsupervised clustering of miRNAs expressed in melanoma cells from the microRNA OSU microarrays (80 samples). (TIFF 1795 kb)

Additional file 3: **Table S1.** Number of Patients with the annotation for the parameter studied in the TCGA cohort. (XLSX 9 kb)

Additional file 4: **Figure S2.** Dispersion plots of KRT5 mRNA with: miR-203, miR-205, MITF, MC1R and MLANA. (JPEG 338 kb)

Additional file 5: **Table S2.** The table showed all the Pearson correlation coefficients of the selected miRNAs with the transcript expression of the selected genes: KRT5, MLANA, and MITF. (XLSX 9 kb)

Additional file 6: **Figure S3.** The mRNA expression of KRT5, KRT6A, KRT6B, KRT6C, MITF, MLANA and BRAF plotted for each sample in melanoma cell lines and patient derived xenografts (PDXs). (TIFF 660 kb)

Additional file 7: **Table S3.** Clinical information of the TCGA cohort. (XLSX 44 kb)

Additional file 8: **Figure S4.** miRNA expression and mutation patterns in melanoma. (JPEG 1133 kb)

Additional file 9: **Figure S5.** Cancer samples plotted according to the somatic mutations and genomic alterations of BRAF, NRAS, NF1, CDKN2A, CCND1 and miR-204 expression. (TIFF 1355 kb)

Additional file 10: **Table S4.** miRNAs and MITF in the most frequent somatic mutations patterns of melanoma (TCGA cohort). (XLSX 9 kb)

Additional file 11: **Table S5.**. Multivariate Cox regression with all the mutational classes took in account for the genomic classification. **Table S6.** Multivariate Cox regression. (XLSX 13 kb)

Additional file 12: **Table S7.** The mutations and miR inhibition for each of the cell lines used in the in vitro experiments. (XLSX 9 kb)

Additional file 13: **Figure S6.** miR-204 loss and melanoma somatic mutations. (TIFF 210 kb)

Additional file 14: **Table S8.** The predicted targets for miR-204 and miR-211: common and miR-specific targets. (XLSX 96 kb)

Additional file 15: Additional methods and data. (PDF 155 kb)

## Abbreviations

AP1S2: Adaptor related protein complex 1 sigma 2
CCND1: Cyclin-D1
CDKN2A: Cyclin-dependent kinase inhibitor 2A
FFPE: Formalin-fixed paraffin blocks
IGFBP5: Insulin like growth factor binding protein
KRT5: Keratin 5
ME: Melanoma metastasis
MITF: Microphthalmia-associated transcription factor
MLANA: Melanoma antigen recognized by T-cells 1
NF1: Neurofibromin 1
OS: Overall survival
PDXs: Patient derived melanoma xenografts
PR: Primary tumors
SERINC3: Serine incorporator 3
SKCM: skin cutaneous melanoma
SOX11: SRY-Related HMG-Box Gene 11
TCGA: The cancer genome atlas
