# Supplementary figures and images for: Loss of miR-204 expression is a key event in melanoma
Source: Mol Cancer. 2018 Mar 9;17:71. doi: 10.1186/s12943-018-0819-8 (PMC5844115; doi:10.1186/s12943-018-0819-8)

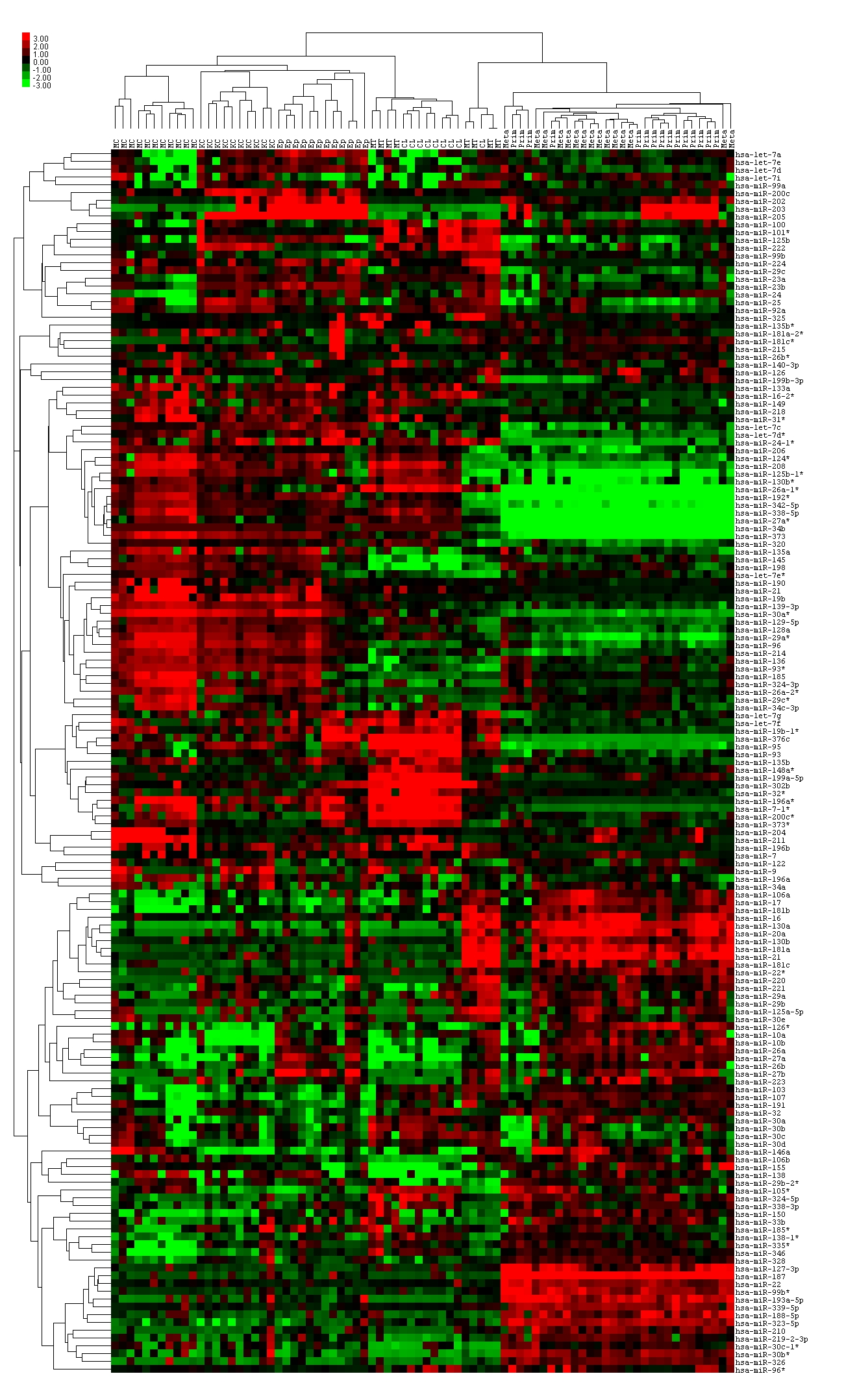

Supplement: Supplementary file 2 — Figure S1. Unsupervised clustering of miRNAs expressed in melanoma cells from the microRNA OSU microarrays (80 samples). (TIFF 1795 kb) [file 12943_2018_819_MOESM2_ESM.tif]

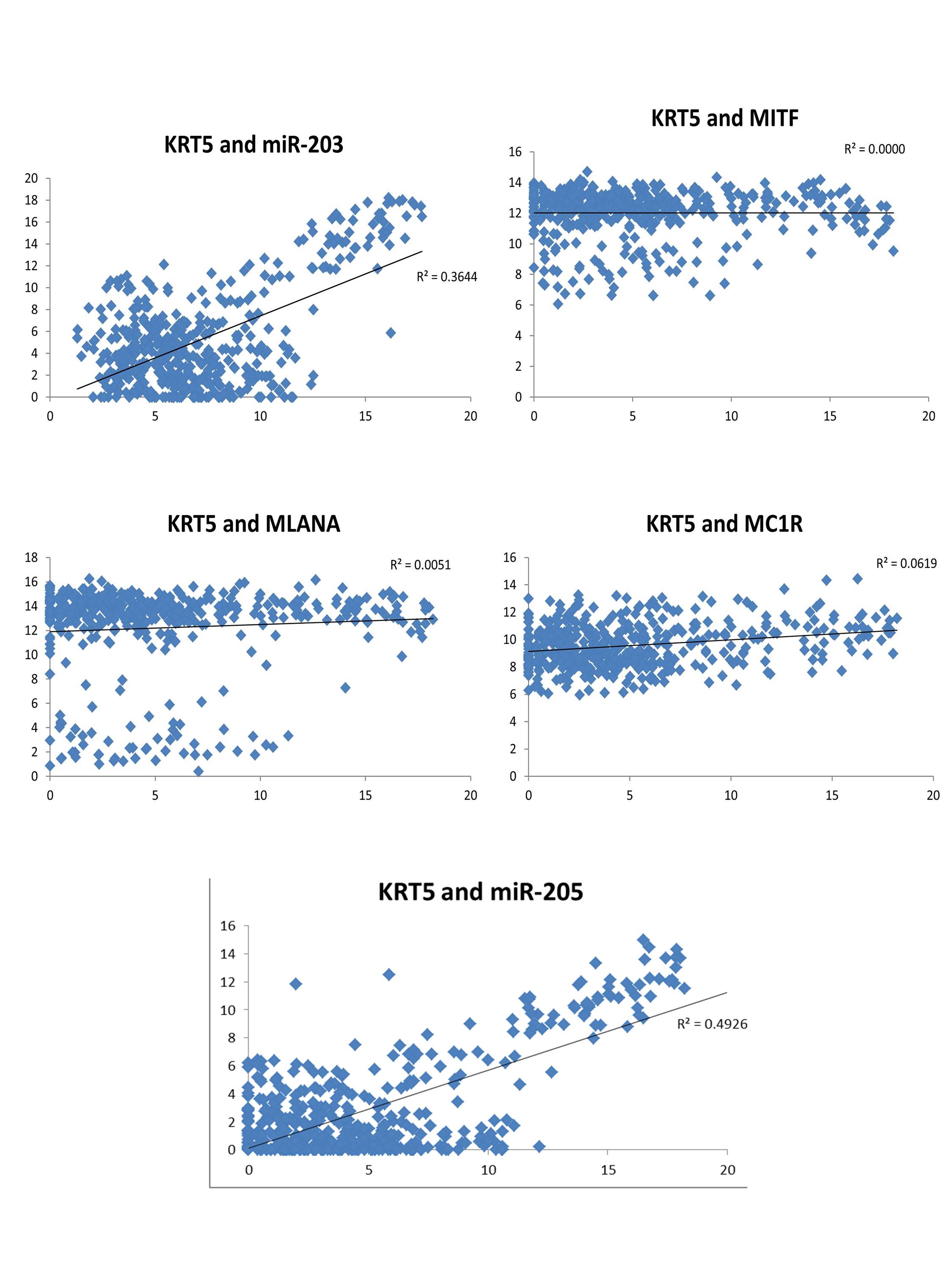

Supplement: Supplementary file 4 — Figure S2. Dispersion plots of KRT5 mRNA with: miR-203, miR-205, MITF, MC1R and MLANA. (JPEG 338 kb) [file 12943_2018_819_MOESM4_ESM.jpg]

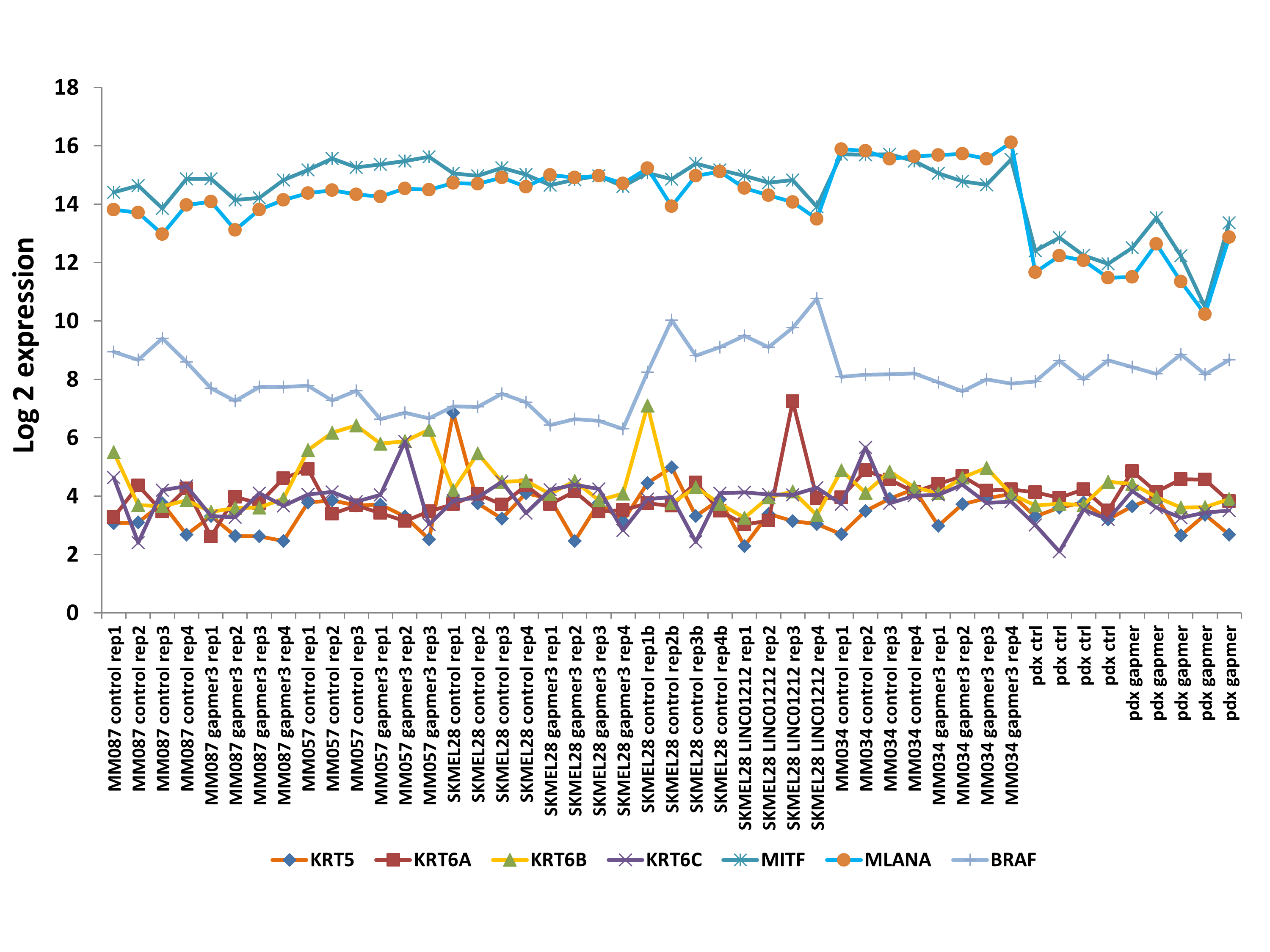

Supplement: Supplementary file 6 — Figure S3. The mRNA expression of KRT5, KRT6A, KRT6B, KRT6C, MITF, MLANA and BRAF plotted for each sample in melanoma cell lines and patient derived xenografts (PDXs). (TIFF 660 kb) [file 12943_2018_819_MOESM6_ESM.tif]

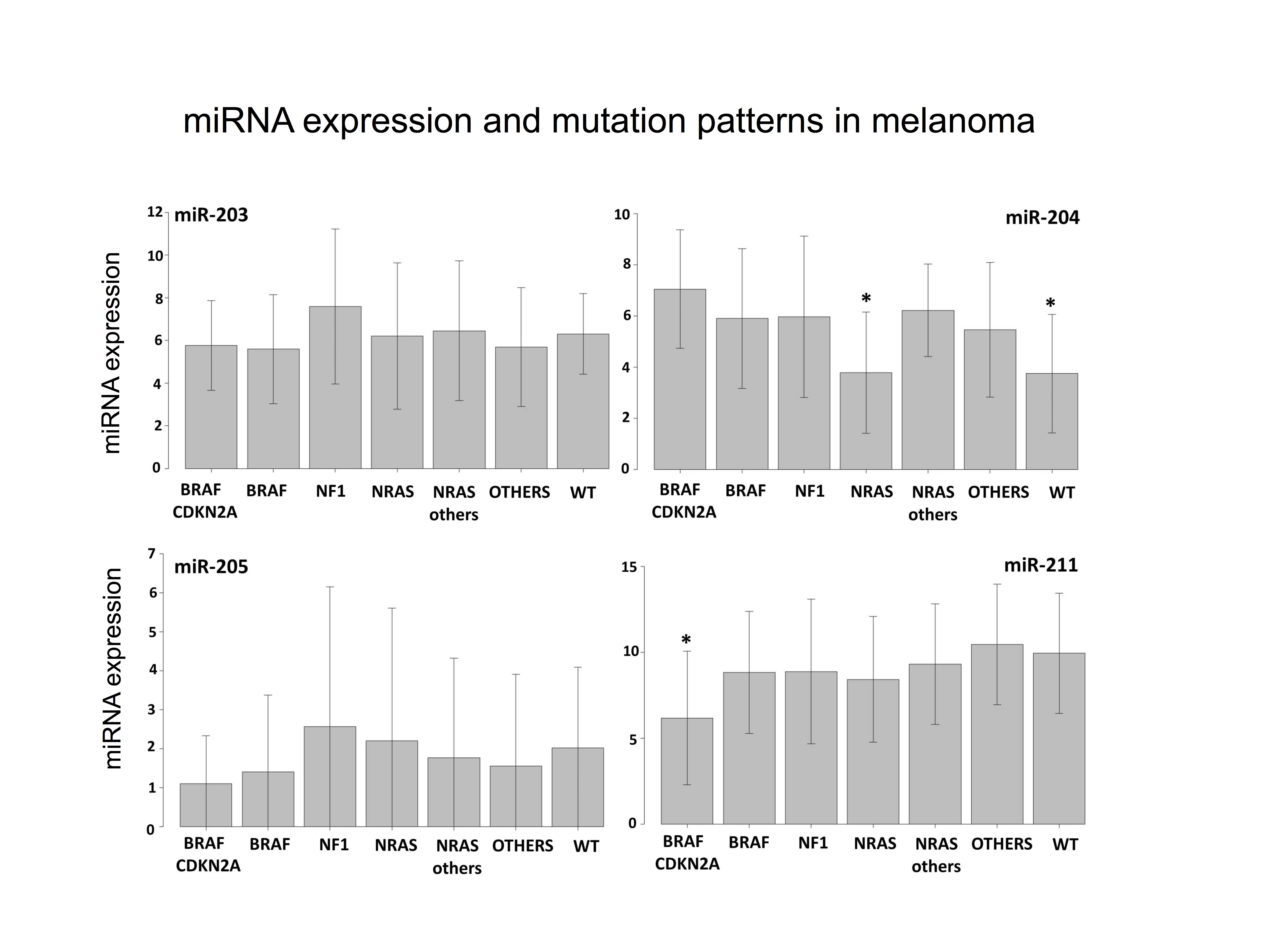

Supplement: Supplementary file 8 — Figure S4. miRNA expression and mutation patterns in melanoma. (JPEG 1133 kb) [file 12943_2018_819_MOESM8_ESM.jpg]

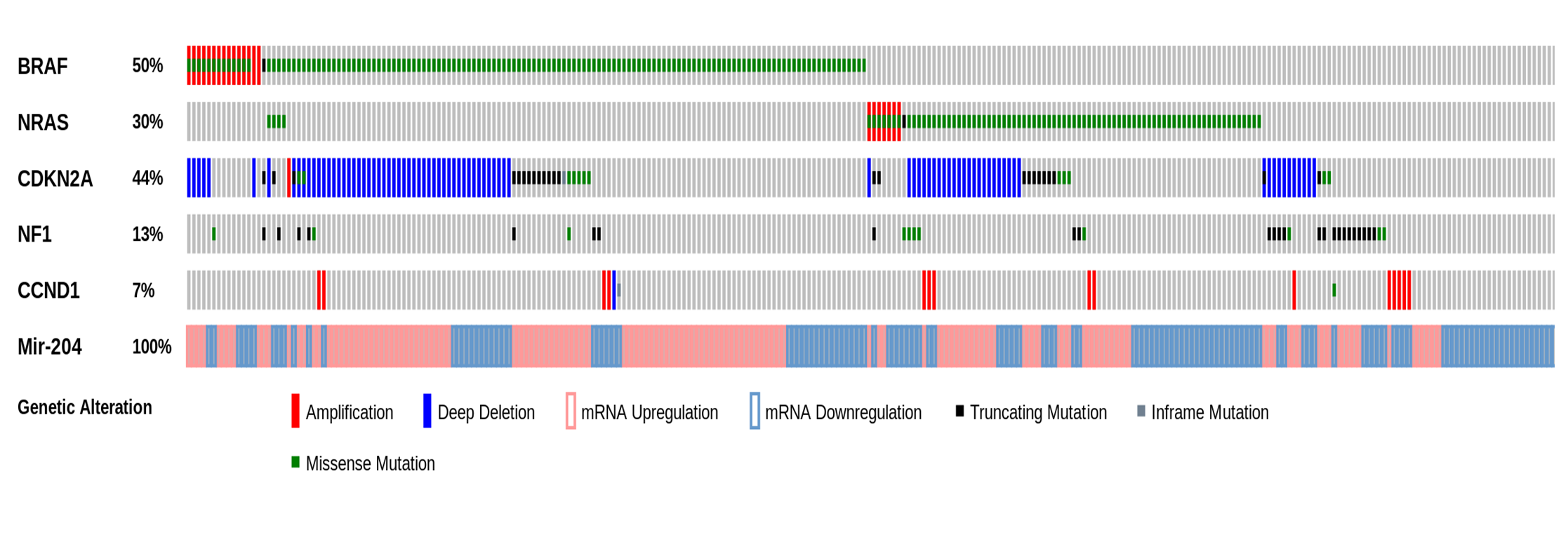

Supplement: Supplementary file 9 — Figure S5. Cancer samples plotted according to the somatic mutations and genomic alterations of BRAF, NRAS, NF1, CDKN2A, CCND1 and miR-204 expression. (TIFF 1355 kb) [file 12943_2018_819_MOESM9_ESM.tif]

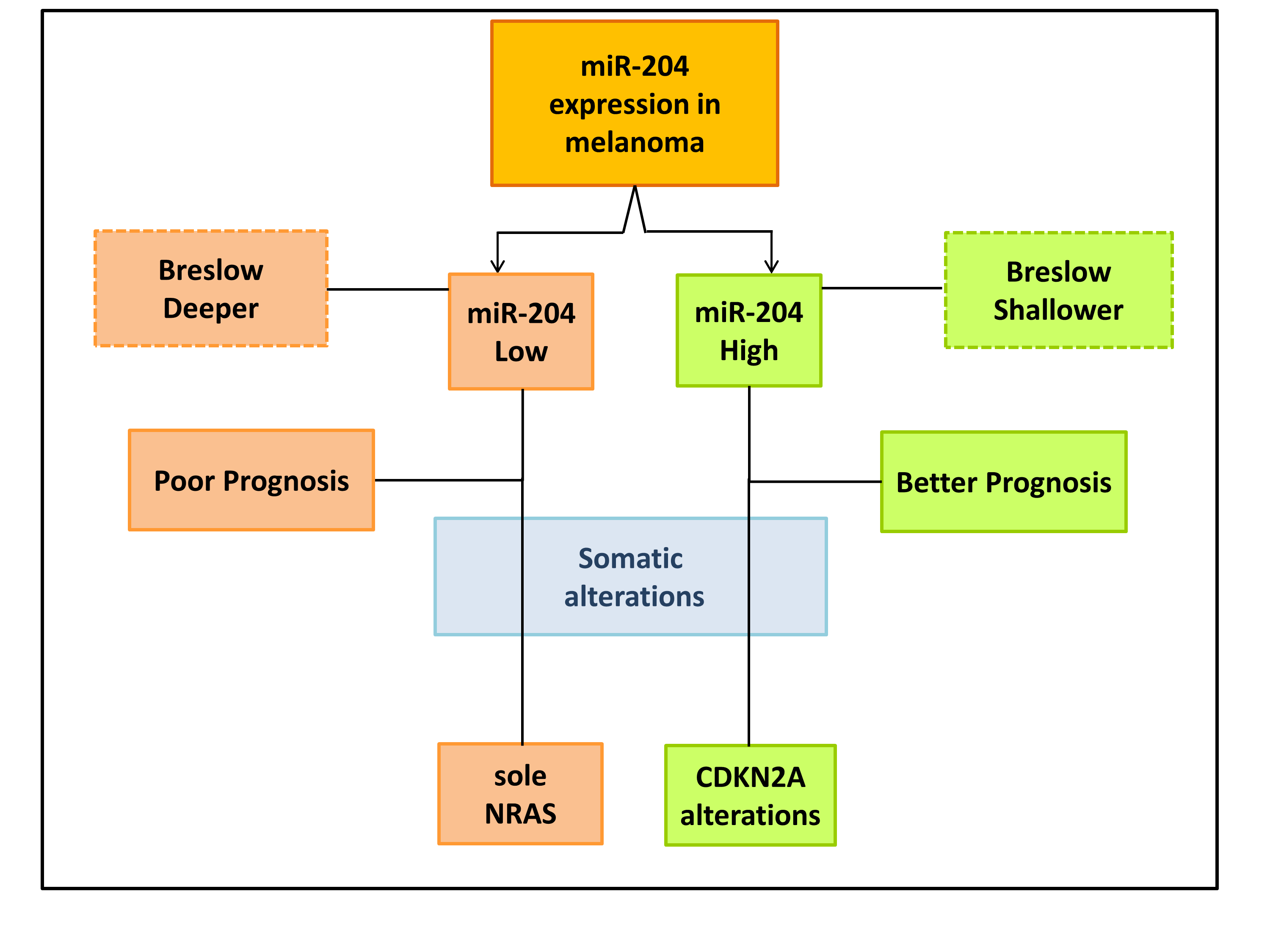

Supplement: Supplementary file 13 — Figure S6. miR-204 loss and melanoma somatic mutations. (TIFF 210 kb) [file 12943_2018_819_MOESM13_ESM.tif]
