# Supplementary material for: Loss of miR-204 expression is a key event in melanoma
Source: Mol Cancer. 2018 Mar 9;17:71. doi: 10.1186/s12943-018-0819-8 (PMC5844115; doi:10.1186/s12943-018-0819-8)
Supplement: Supplementary file 15 — Additional methods and data. (PDF 155 kb) [file 12943_2018_819_MOESM15_ESM.pdf]

ADDITIONAL METHODS AND DATA

Methods

Samples and miRNA expression. Eighty samples, including 15 pairs of matched primary/metastatic tumours, 12 normal skin biopsies, 11 cultured melanocytes, 10 cultured keratinocytes, 17 metastatic melanoma cell lines (7 short term "MT" and 10 long term "CL") were analyzed on miRNA microarrays, as previously reported [1]. The table with quantile normalized miRNA expression is reported in Additional File 1. The data for mature miRNA expression were confirmed by stem loop RT-PCR, according to the manufacturer protocol (ABI, Foster City, CA). One nanogram of total RNA was assayed and U6 non-coding RNA was used as normalizer. Curative surgery was undertaken in all patients. Patients with melanoma were chemotherapy naïve. The primary melanocytes were derived from neonatal foreskin. Less than 1% contaminant cell types (i.e. keratinocytes, lymphocytes, granulocytes) were present in these cultures. Early passage or short-term melanoma cell cultures and long term melanoma cell lines were derived from metastatic melanoma tumors[2]. Cell lines were maintained in 10% FCS and RPMI. The early passage cell lines were maintained in culture for less than 30 passages, and their composition was for more than 99% melanoma cells. The OSU cohort included formalin-fixed paraffin block (FFPE) samples (n=30), from primary and metastatic paired tumors. Three 2-mm cores were obtained from the micro-dissected FFPE for the melanoma matched primary/metastatic tumors. Within metastatic melanoma lesions, there may be an admixture of infiltrating lymphocytes (and lesser numbers of macrophages, dendritic cells, fibroblasts and endothelial cells), therefore prior to RNA extraction two pathologists verified that the majority of cells in the melanoma tumors were malignant (>90%). Total RNA extraction was undertaken using the RecoverAll kit (Ambion Inc, Austin, Tex) according to manufacturer's instructions.

Cell lines, miRNA transfections and Western Blots. MDA-MB-435, G361 and VAG, COLO-38, Me-1007 cells were cultured respectively in Dulbecco's modified Eagle's medium DMEM (GE-Healthcare), McCoy's 5A Medium (Sigma) and RPMI 1640 (Sigma) supplemented with 10% FBS, L-glutamine, and penicillin/streptomycin. Transfections with 100 nM of miR-204-5p and miR-211-5p, miR negative control (Ambion), anti-miR-204-5p, and anti-miR-211-5p (Fidelity Systems Inc.) RNA oligonucleotides, were performed using siPORT (Life Technologies). 150.000 cells/well were plated in 12-well plates and after 16 hours (h) the complete medium was replaced with 0.1% FBS. Then, cells were transfected and maintained in 0.1% FBS medium for further 24h. The cells were finally harvested 6h after 10% FBS replacement. The experiments were performed in triplicate and/or quadruplicate. Viability in cell culture was assayed with MTS at time different intervals, in accordance with manufacturer's instructions (Promega Corp., Madison, WI). MTS results were showed as cell growth inhibition percentage relative to the control sample.

Validation cohorts. miRNA and mRNA expression data and the corresponding clinical information for the skin cutaneous melanoma (SKCM) dataset were obtained from The Cancer Genome Atlas (TCGA) data portal (https://cancergenome.nih.gov). The level 3 data (reads count) for the RNA patients were quantile normalized and log2 transformed. From the SKCM dataset, we selected samples with miRNA, mRNA and overall survival (OS) data. Patients showing OS shorter than 30 days were excluded from the analysis. The expression study with melanoma cell lines and patient derived xenografts (PDXs) was obtained from GEO (accession GSE70180). Data were analyzed using BRB ArrayTools (https://brb.nci.nih.gov/BRB-ArrayTools), R (https://www.r-project.org), BioConductor (https://www.bioconductor.org) and IBM SPSS Statistics.

Statistics. Paired t-test was used to assess the relative expression levels ($2^{-\Delta Ct}$) in cell lines derived from primary and metastatic melanomas. The correlations between the expression levels of miRNA and mRNA were calculated using Pearson correlation. The relationships between miRNA expression and

clinical variables were determined using Spearman correlation. Survival analysis was performed using the Kaplan-Meier method and statistics were based on log-rank testing. A multivariable Cox proportional hazard model was used, coupled with backward stepwise selection, to identify miRNAs with independent prognostic value. Two tails t-test was used to compare the means and $P < 0.05$ were considered significant; FDR and the Holm method were used to account for multiple testing. Statistical analyses were performed using BRB-Array Tools, R/BioConductor, and SPSS (v21). To verify the existence of a statistically significant trend in miR-211 and miR-204 expression consistent with melanoma invasiveness grade, a Jonckheere-Terpstra two-tailed trend test was used on TCGA patients: thus we were able to identify a positive trend between Breslow's stage and miR-211 expression levels (n = 220, trend coefficient = 2.27, P-value = 0.023), a positive trend between Clark's and miR-211 levels (n = 212, trend coefficient = 2.45, P-value = 0.014) and a negative trend between Breslow's stage and miR-204 levels (n = 212, trend coefficient = -2.46, P-value = 0.014).

List of Additional Tables

Additional Table 1. Patients with annotation for the clinical parameters in the TCGA cohort.

Additional Table 2. The Pearson correlation coefficients of the selected miRNAs with the transcript expression of protein coding genes: KRT5, MLANA, and MITF.

Additional Table 3. Clinical information for the samples used from TCGA cohort.

Additional Table 4. miRNAs and MITF in the most frequent somatic mutations patterns of melanoma (TCGA cohort).

Additional Table 5. Multivariable Cox regression with the mutational subclasses studied in the genomic classification.

Additional Table 6. Multivariable Cox regression with the BRAF and NRAS mutations in each patients.

Additional Table 7. The mutation and miRNA inhibition report for the cell lines used *in vitro*.

Additional Table 8. The predicted targets for miR-204 and miR-211: common and miR-specific targets.

List of Additional Figures

Additional Figure 1. Unsupervised clustering of miRNAs expressed in melanoma cells from the microRNA OSU microarrays (80 samples).

Additional Figure 2. Dispersion plots of KRT5 mRNA with: miR-203, miR-205, MITF, MC1R and MLANA.

Additional Figure 3. The mRNA expression of KRT5, KRT6A, KRT6B, KRT6C, MITF, MLANA and BRAF plotted for each sample in melanoma cell lines and patient derived xenografts (PDXs).

Additional Figure 4. Bar plots showing miRNAs expression in relation with defined somatic alterations.

Additional Figure 5. Cancer samples plotted according to the somatic mutations and genomic alterations of BRAF, NRAS, NF1, CDKN2A, CCND1 and miR-204 expression.

Additional Figure 6. miR-204 loss and melanoma somatic mutations.

Additional Figures Legend

Additional Figure 1
To identify miRNAs expressed in melanoma we applied the microRNA OSU microarray platform to 80 samples including 11 cultured melanocytes, 10 cultured keratinocytes, 12 normal skin biopsies, 17 melanoma cell lines and 15 pairs of matched primary/metastatic melanoma biopsies. Using unsupervised analysis, we identified 157 highly variable miRNAs. Unsupervised clustering clearly showed the differential distribution of miRNA expression in the different cell types: epidermis (EP), normal cultured keratinocytes (KC), normal cultured melanocytes (MC), short (MT) and long term (CL) melanoma cell lines (*f-test,* $P < 0.001$). Average linkage clustering was performed by using un-centered correlation metric.

Additional Figure 2
Dispersion plots showing that Keratin 5 (KRT5) mRNA, miR-203 and miR-205 were highly correlated (Pearson r=0.613 and r= 0.771, $P < 1e\text{-}07$) in the TCGA samples. MITF, MC1R and MLANA bear no significant correlation with KRT5.

Additional Figure 3
The mRNA expression of keratins (KRT5, KRT6A, KRT6B and KRT6C), MITF, MLANA and BRAF plotted for each sample in melanoma cell lines and patient derived xenografts (PDXs). On the *y-axes* the log2 expression values are reported.

Additional Figure 4
Bar plots showing miRNAs expression in relation with somatic alterations. Cancer samples were split into 7 groups according to their mutation patterns: sole BRAF (n=57), sole NRAS (n=37), sole NF1 (n=18), BRAF and CDKN2A (n=59), non-sole NRAS (n=42), remaining mutations (n=32), and no somatic events in any of the above-mentioned genes (n=29). miR-205 and miR-203 were not associated with any mutation. miR-204 and miR-211 were significantly (star labelled) associated with somatic mutations, and with different trends.

Additional Figure 5
Cancer samples (n=274) were plotted using OncoPrinter (http://www.cbioportal.org) according to the somatic mutations and genomic alterations of BRAF, NRAS, NF1, CDKN2A, CCND1. At the bottom of the panel the miR-204 expression is reported, as high (red) or low (blue).

Additional Figure 6
miR-204 loss and melanoma somatic mutations. The miR-204 expression levels in melanoma samples, "Low" (light orange) or "High" (light green), were defined splitting the cohort by the median level. Breslow's thickness and Overall Survival (OS) were associated with miR-204 expression. miR-204 loss was preferentially associated with sole NRAS mutation or wild type CDKN2A melanoma genotypes.

SUPPLEMENTAL REFERENCES

1. Volinia S, Calin GA, Liu CG, Ambs S, Cimmino A, Petrocca F, Visone R, Iorio M, Roldo C, Ferracin M *et al*: **A microRNA expression signature of human solid tumors defines cancer gene targets**. *Proc Natl Acad Sci U S A* 2006, **103**(7):2257-2261.

2. Spath L, Ulivieri A, Lavra L, Fidanza L, Carlesimo M, Giubettini M, Narcisi A, Luciani E, Bucci B, Pisani D *et al*: **Antiproliferative Effects of 1alpha-OH-vitD3 in Malignant Melanoma: Potential Therapeutic implications**. *Sci Rep* 2017, **7**:40370
